# Pediatric Pes Planovalgus and Femoral Antetorsion: Understanding a Biomechanical Unit: A Narrative Review of the Proximal-to-Distal Kinetic Chain in Childhood Flatfoot

**DOI:** 10.3390/children13040510

**Published:** 2026-04-06

**Authors:** Carlo Camathias, Victor Valderrabano, Erich Rutz, Bernhard M. Speth

**Affiliations:** 1 Praxis Momentum, Brauerstrasse 97, CH-9016 St. Gallen, Switzerland; 2 Medical Faculty, University of Basel, CH-4056 Basel, Switzerland; 3 Swiss Ortho Center, Swiss Medical Network, Schmerzklinik Basel, Hirschgässlein 15, CH-4010 Basel, Switzerland; 4 Department of Paediatric Orthopaedic Surgery, The Royal Children’s Hospital Parkville, Melbourne 3052, Australia; 5 Kinder- und Jugendorthopädie, KSA am Bahnhof, Bahnhofplatz 3c, CH-5000 Aarau, Switzerland

**Keywords:** pes planovalgus, flexible flatfoot, femoral antetorsion, rotational profile, pediatric or-thopedics, biomechanics, spontaneous correction, derotation osteotomy

## Abstract

**Highlights:**

**What are the main findings?**

- **Strong statistical association exists** between pediatric pes planovalgus and increased femoral antetorsion in preschool children (r = 0.53–0.77), though this correlation weakens with age and direct causation remains unproven.
- **Parallel developmental trajectories** characterize both deformities: femoral antetorsion decreases from ~35° at age three to 10–15° in adulthood, while flatfoot prevalence drops from 44 to 54% at ages 3–4 to under 15% by age 10, with critical correction occurring before age eight.

**What are the implications of the main findings?**

- **Watchful waiting is justified** for flexible, asymptomatic flatfoot in preschool children (ages 3–6), as both femoral antetorsion and flatfoot undergo spontaneous correction during normal development without intervention.
- **Comprehensive rotational assessment** (including tibial torsion and foot progression angle) should precede any surgical intervention in persistent symptomatic cases, as the relationship between proximal femoral geometry and distal foot alignment represents a complex three-dimensional phenomenon rather than a simple mechanical cascade.

**Abstract:**

**Background:** Pes planovalgus affects 44–54% of preschool children and represents one of the most common concerns in pediatric orthopedic practice. **Aim:** This narrative review synthesizes the evidence linking increased femoral antetorsion to pediatric flatfoot deformity. **Methods:** A comprehensive literature search was conducted in PubMed, Scopus, and Web of Science through January 2026. The initial search yielded 847 records; after screening, 52 studies were included, 29 of which are directly cited. Search terms included combinations of: “femoral antetorsion” OR “femoral anteversion” AND “flatfoot” OR “pes planovalgus” AND “children” OR “pediatric”. **Results:** Strong correlations exist between flatfoot and increased internal hip rotation (as a proxy for femoral antetorsion) in preschool children (r = 0.53–0.77), suggesting an association, though direct causation remains unproven. Both deformities share similar developmental trajectories with spontaneous resolution by school age. The biomechanical model proposes that elevated antetorsion reduces gluteus medius moment arms by 40–50%, necessitating compensatory hip internal rotation; however, this derives from computational models and cerebral palsy populations, with limited direct validation in typically developing children. Femoral derotation osteotomy improves the foot progression angle, though transfer efficiency is incomplete (~54% of surgical correction manifests distally). **Conclusions:** Femoral antetorsion and pes planovalgus are strongly associated in preschool children, though whether this represents a direct mechanistic cascade or parallel manifestations of common developmental factors remains uncertain. This understanding supports watchful waiting in preschool children and, in persistent cases, prioritizes the assessment of the entire rotational profile before intervention.

## 1. Introduction

Few conditions in pediatric orthopedics generate as much parental anxiety—and as many unnecessary interventions—as the flexible flatfoot. The sight of a toddler’s feet rolling inward during weight-bearing triggers concerns about future disability, prompting consultations, shoe modifications, and orthotic prescriptions despite limited and inconsistent evidence for their efficacy [1,2,3,4]. Yet this focus on the foot as an isolated anatomical unit overlooks a fundamental biomechanical truth: the lower extremity functions as an integrated kinetic chain, where the geometry of the proximal femur profoundly influences alignment and loading patterns all the way down to the toes.

The relationship between increased femoral antetorsion and pediatric flatfoot represents one of the most robust—and most clinically underappreciated—associations in pediatric orthopedics. In two landmark studies of preschool children, researchers found that all children with flatfoot demonstrated elevated internal hip rotation, with correlation coefficients of r = 0.53–0.77 [5,6].

These landmark studies employed clinical hip rotation measurements as a proxy for femoral antetorsion rather than direct radiographic measurements (CT/MRI). Clinical and radiographic measures may diverge by 15–20 degrees, and substantial overlap exists between flatfoot and non-flatfoot populations (mean internal rotation 55.8° vs. 53.0°), suggesting limited diagnostic specificity [7]. Furthermore, the age-dependent weakening of this correlation in older children [7]. raises the question of whether these represent mechanistically linked deformities or parallel manifestations of common developmental factors, such as generalized ligamentous laxity or neuromuscular immaturity.

Perhaps most remarkably, both conditions share nearly identical developmental trajectories. Femoral antetorsion decreases from approximately 35° at age three to 10–15° in adulthood, with the critical correction window occurring before age eight [8,9,10]. The medial longitudinal arch develops in parallel, with flatfoot prevalence declining from approximately 44–54% at ages 3–4 to under 15% by age 10 [1,11,12]. 

The purpose of this narrative review is to synthesize the epidemiological, biomechanical, and clinical evidence examining the association between femoral antetorsion and pes planovalgus during childhood development, while acknowledging current limitations in establishing direct causation.

## 2. Materials and Methods

### 2.1. Search Strategy

This narrative review was conducted following PRISMA guidelines where applicable. We performed systematic searches of PubMed/MEDLINE, Scopus, and Web of Science databases from inception through January 2026. Search terms included combinations of: “femoral antetorsion” OR “femoral anteversion” AND “flatfoot” OR “pes planovalgus” AND “children” OR “pediatric”.

**Figure 1:**
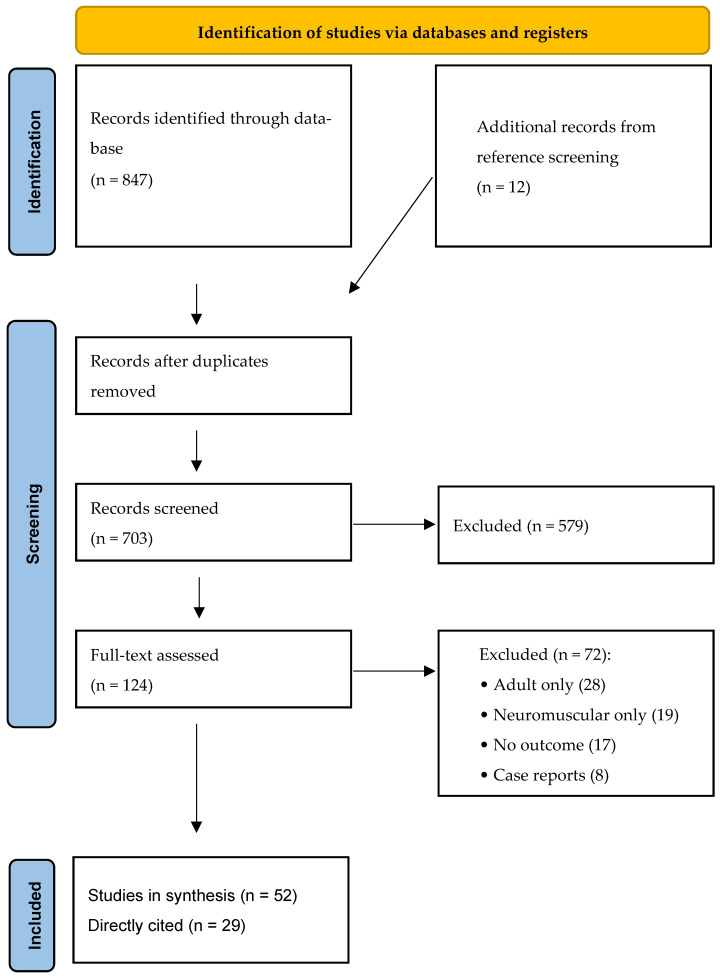
PRISMA flow diagram of study selection.

The initial search yielded 847 records. After then removal of duplicates (n = 144) and screening, 124 full-text articles were assessed for eligibility. Of these, 52 studies met the inclusion criteria. Reference lists were manually screened, yielding 12 additional sources. A total of 29 studies are directly cited (Figure 1).

### 2.2. Eligibility Criteria

Studies were included if they: (1) examined the correlation between femoral version and foot posture in children; (2) analyzed the biomechanical mechanisms linking hip rotation to foot loading; (3) reported natural history data; (4) evaluated treatment outcomes for femoral derotation osteotomy; or (5) provided clinical guidelines. Case reports and adult-only studies were excluded.

## 3. Results

### 3.1. Epidemiological Evidence

The landmark study by Zafiropoulos et al. (2009) [5] prospectively examined 651 Greek children aged 3–6 years. The critical finding: every single child with flatfoot demonstrated elevated internal hip rotation. The correlation coefficient was r = 0.53 (*p* < 0.001), with mean internal rotation reaching 69.9° in the flatfoot children (Table 1).

Jemni et al. (2015) [6] confirmed these findings in a Tunisian cohort of 110 children with r = 0.73–0.77 (*p* < 0.001). Once again, all flatfoot children demonstrated elevated femoral antetorsion. Notably, approximately 80% also showed insufficient external tibial torsion, suggesting that foot deformity may be associated with a complex three-dimensional rotational malalignment of the entire lower extremity, not solely due to femoral antetorsion.

Importantly, Žukauskas et al. (2021) [7] found no significant correlation between flatfoot indicators and hip rotation in 301 children aged 5–8 years (mean internal rotation: 55.8° in flatfoot vs. 53.0° in children without flatfoot). This age-dependent weakening of the association—with substantial overlap between groups—suggests that both conditions undergo parallel spontaneous correction during the preschool years. Whether this reflects mechanical interdependence or the independent maturation of shared developmental factors remains unclear. [13].

### 3.2. The Biomechanical Model

Arnold, Komattu, and Delp (1997) [14] established the biomechanical rationale using 3D computer modeling: femoral antetorsion exceeding 30° reduces the gluteus medius moment arm by 40–50%. Approximately 30° of compensatory internal rotation restores the moment arm to within 5% of normal. However, this model derives from adult computational simulations and has not been directly validated in typically developing children.

De Pieri et al. (2023) [15] confirmed reduced gluteus medius abductive contribution during gait in patients with elevated antetorsion (39.6° ± 6.9° vs. 18.7° controls), providing empirical support for the theoretical model.

Chang et al. (2002) [16] demonstrated that dynamic pedobarography can objectively document varus and valgus foot deformities in children with cerebral palsy, establishing a coronal index that correlates highly with clinical assessment. Their work showed that medial versus lateral foot loading patterns directly reflect the severity of hindfoot malalignment. Importantly, the Chang et al. study does not directly demonstrate that rotational changes at the femoral level influence foot loading distribution; rather, it establishes pedobarographic methodology for documenting foot deformities in CP. The inference that proximal femoral rotation affects distal foot loading patterns remains an extrapolation from indirect evidence, and direct validation in typically developing children is currently lacking. Furthermore, these data derive from a neurologically impaired population where muscle tone and motor control differ fundamentally from typically developing children.

Limitations of the Mechanistic Model. While the Arnold model elegantly explains how increased antetorsion reduces gluteus medius moment arms, several limitations warrant consideration. First, this derives from adult computational models and has not been validated in normally developing children. Second, the weak correlation between derotation magnitude and foot progression angle improvement suggests incomplete proximal-to-distal transfer: Naqvi (2017) reported 40.8° mean surgical derotation but only ~22° of FPA change [17]—approximately 54% transfer efficiency [18]. The remaining correction may be absorbed by compensatory changes at the pelvis, knee, or tibia. Third, the finding that 80% of flatfoot children exhibit insufficient external tibial torsion [6] indicates that foot deformity is a complex three-dimensional malalignment, not solely a consequence of femoral antetorsion. The proposed proximal-to-distal cascade may oversimplify this relationship.

### 3.3. Developmental Biology

Fabry, MacEwen, and Shands (1973) [10] established the critical window: femoral antetorsion decreases progressively, but no significant decrease occurs after age eight. Staheli’s survey of 882 feet [11] documented parallel arch development, with flatfoot prevalence dropping from nearly 100% at age two to approximately 4% by age 10 (Table 2).

The mechanisms underlying parallel spontaneous correction of antetorsion and flatfoot remain poorly understood. Possibilities include bone remodeling in response to mechanical loading, maturation of ligamentous support, development of neuromuscular control, and growth-related geometric changes. Prospective imaging studies correlating these parameters are lacking.

### 3.4. Therapeutic Implications

***Watchful Waiting***

The German S2k guideline (AWMF 187-053) [19] states: “Flexible painless physiologic flatfoot should NOT be specifically treated, including in children under six years.” This recommendation for watchful waiting applies equally to both femoral antetorsion and pes planovalgus in the 3–6 year age group, as both conditions undergo spontaneous correction during this developmental period [8,10,11]. Consequently, watchful waiting encompasses the entire proximal-to-distal kinetic chain, not just the foot deformity in isolation.

***Conservative Treatment: Neither Foot Orthoses Nor Non-Operative Methods Alter Underlying Anatomy***

Two distinct bodies of evidence converge on this conclusion. Regarding femoral antetorsion specifically, Fabry et al. [20] demonstrated that non-operative methods—including shoe wedges, twister cables, and night splints—”do not appear effective” for reducing femoral antetorsion. Regarding foot-directed interventions separately, the Cochrane review by Evans et al. (2022) [3] concluded that one “cannot conclude” that foot orthoses are effective for pediatric flatfoot, and Wenger et al. (1989) [4,21] showed that all groups improved spontaneously regardless of whether corrective shoes, inserts, or no treatment was provided. Thus, conservative approaches cannot correct even the foot deformity itself, let alone influence proximal femoral geometry.

***Surgical Intervention When Necessary***

Naqvi et al. (2017) [18] reported that in 35 neurologically typical limbs (mean age 13.3 years, range 8–18), foot progression angle improved from approximately −15° (internal) to +8° (external) after femoral derotation osteotomy (*p* < 0.0001). Long-term studies in cerebral palsy populations [22,23] confirmed stability over 5–11 years. However, these surgical series lack matched non-operative control groups, precluding determination of whether observed improvements exceed natural history. Furthermore, the applicability of data from school-age children (mean 13.3 years) to the preschool population with physiologic flatfoot remains uncertain (Table 3).

***Foot-Directed Surgery for Isolated Foot Pathology***

For isolated symptomatic valgus foot deformity without significant femoral antetorsion, foot-directed surgical procedures are appropriate. Established options include calcaneal lengthening osteotomy (Evans/Mosca procedure) [24,25], medial displacement calcaneal osteotomy, or subtalar arthroereisis. The choice of procedure depends on the severity of the deformity, the child’s age, and the specific anatomical findings. These foot-directed interventions address the distal pathology directly and do not require concurrent proximal correction when femoral geometry is within normal limits.

## 4. Discussion

The correlation coefficients of r = 0.53–0.77 observed in preschool children [5,6] demonstrate a strong statistical association between flatfoot and increased hip internal rotation. However, correlation does not establish causation. The age-dependent weakening of this correlation [7] suggests that femoral antetorsion and flatfoot may be parallel manifestations of a common developmental factor—such as generalized ligamentous laxity or neuromuscular immaturity—rather than a direct mechanistic cascade where one causes the other.

While all flatfoot children in the landmark studies demonstrated elevated internal rotation, the overlap with non-flatfoot populations is substantial (mean difference ~2–3°), suggesting limited specificity as a diagnostic criterion [7]. This overlap, combined with the use of clinical rotation as a proxy for radiographic antetorsion, means that the reported “100% co-occurrence” should be interpreted cautiously.

For clinicians, we propose a practical framework:

**Ages 3–6:** No treatment for flexible, asymptomatic flatfoot. Document the complete rotational profile (femoral antetorsion, tibial torsion, foot progression angle) and counsel parents regarding the expected favorable natural history. Orthoses are not indicated [3,4,19]. At this age, clinical examination is sufficient and preferred over imaging to avoid radiation exposure. Femoral antetorsion is estimated via the prone internal/external hip rotation test (with the hip extended and the knee flexed to 90°). Tibial torsion is assessed using the thigh–foot angle with the child prone and the knee flexed to 90°. The foot progression angle can be assessed clinically by observing the gait pattern (in-toeing versus out-toeing) or measured as the angle between the foot axis and the line of progression during walking. Formal instrumented gait analysis is not necessary at this stage. We recommend monitoring at 6–12 month intervals until age eight.

**Ages 7–10:** Continue observation if the condition is improving. Symptomatic flatfoot may benefit from sensomotor insoles for comfort only—these do not alter the underlying anatomy. Sensomotor (proprioceptive) insoles are custom-molded orthoses with specific elevations and depressions designed to stimulate plantar mechanoreceptors, aiming to improve neuromuscular foot control rather than passively supporting the arch. This distinguishes them from traditional rigid arch supports; they may improve comfort and proprioceptive feedback but cannot alter the underlying skeletal geometry of either the foot or the proximal femur. [26].

**Age > 10:** Persistent symptomatic deformity warrants a comprehensive evaluation. If significant femoral antetorsion persists (antetorsion > 30°, internal hip rotation > 70°, external rotation < 20°), proximal correction via derotation osteotomy may be considered, recognizing that transfer efficiency to the foot is incomplete (~54%) and that tibial torsion abnormalities may independently contribute to foot malalignment. Indications for femoral derotation osteotomy extend beyond isolated in-toeing and include functional impairment (recurrent tripping, activity limitation), anterior knee pain or patellar instability secondary to rotational malalignment [27,28], and significant cosmetic or psychological distress to the child or adolescent. Isolated foot pain without significant femoral antetorsion would not warrant proximal correction. For isolated symptomatic valgus foot deformity without significant antetorsion, foot-directed procedures, such as calcaneal lengthening osteotomy (Evans/Mosca procedure), medial displacement calcaneal osteotomy, or subtalar arthroereisis, are appropriate [24,25]. Three-dimensional instrumented gait analysis, when available and feasible, can be valuable for preoperative planning in persistent cases, as it objectively quantifies the individual contribution of femoral antetorsion, tibial torsion, and foot deformity to the overall rotational malalignment [29].

### Limitations

Several significant limitations warrant consideration. First, landmark correlation studies employed clinical hip internal rotation as a proxy for femoral antetorsion rather than direct radiographic measurement (CT/MRI). Clinical hip rotation and radiographic antetorsion can diverge by 15–20 degrees, and the overlap between flatfoot and non-flatfoot populations is substantial (~2–3° mean difference in internal rotation), limiting diagnostic specificity.

Second, the biomechanical model derives from adult computational modeling [14] and cerebral palsy populations [16]; direct validation in normally developing children is absent. The proposed proximal-to-distal cascade, while plausible, remains largely hypothetical in typically developing children.

Third, surgical outcome data [18,19,20,21,22,23] lack matched non-operative control groups, precluding determination of whether observed improvements exceed natural history. Furthermore, the mean age of surgical patients (13.3 years in Naqvi [18]) differs substantially from the preschool population where the correlation was established.

Fourth, the three-dimensional nature of rotational malalignment—with 80% of flatfoot children showing concomitant tibial torsion abnormalities [6]—is underexplored. Focusing solely on femoral antetorsion may oversimplify the pathomechanism. Moreover, the various biomechanical models cited in this review only weakly consider tibial torsion, despite the tibia being the anatomical link between the femur and the foot; future models should integrate tibial torsion more explicitly to better represent the complete rotational chain. Three-dimensional instrumented gait analysis can help delineate the individual contributions of each level of the rotational profile; however, availability, cost, and the requirement for child cooperation limit its routine use, particularly in preschool-age children.

Finally, potential confounders such as gender (flatfoot prevalence: boys 52%, girls 36% [1]) and generalized ligamentous laxity [30] have not been adequately controlled in correlation studies. Future research should investigate whether the association between antetorsion and flatfoot is gender dependent.

## 5. Conclusions

Strong correlations exist between pediatric pes planovalgus and increased femoral antetorsion in preschool children (r = 0.53–0.77), suggesting association, though direct causation remains unproven. Both deformities share similar developmental trajectories, with spontaneous resolution by school age. Whether this represents a direct mechanistic cascade or parallel manifestations of common developmental factors (e.g., ligamentous laxity) remains uncertain. In preschool children, the cornerstone of treatment is parental education and reassurance. For the minority with persistent symptomatic deformity, clinicians should assess the entire rotational profile—including tibial torsion—before considering intervention. When femoral derotation is performed, incomplete transfer to the foot (~54%) and potential contribution of tibial malalignment should be anticipated.

## Figures and Tables

**Table 1:** Key correlation studies.

Study	n	Age	r	Key Finding
Zafiropoulos 2009 [5]	651	3–6 y	0.53 ***	100% flatfoot had ↑ IR (69.9°)
Jemni 2015 [6]	110	3–6 y	0.73–0.77 ***	100% ↑ FAT; 80% ↓ ext. tibial torsion
Žukauskas 2021 [7]	301	5–8 y	NS	No correlation; overlap (55.8° vs. 53.0°)

*** *p* < 0.001; NS = not significant; IR = internal rotation; FAT = femoral antetorsion; ↑ = increase; ↓ = decrease.

**Table 2:** Parallel developmental trajectories [4,5,15,16].

Age	Femoral Antetorsion	Flatfoot Prevalence	Implication
Birth	30–40°	~100%	Physiological
2–3 years	~30–35°	~45–55%	Peak presentation
4–6 years	~25–30°	~25–40%	Correction begins
7–10 years	~15–20°	~4–15%	Near-complete
Adult	8–14°	15–23%	Final values

**Table 3:** Foot progression angle changes after femoral derotation osteotomy.

Study	n	Population	FPA Pre	FPA Post	Follow-Up
Naqvi 2017 [18]	35	Normal (13.3 y)	−15.2°	+7.7°	2 years
Ounpuu 2002 [22]	27	CP	~−5°	~−12°	5 years
Õunpuu 2017 [23]	27	CP	~−9°	~+13°	11 years

FPA = foot progression angle; CP = cerebral palsy; negative values indicate internal rotation.

## Data Availability

Not applicable.

## Abbreviations

The following abbreviations are used in this manuscript:

**Abbreviation**	**Definition**
AWMF	Arbeitsgemeinschaft der Wissenschaftlichen Medizinischen Fachgesellschaften
CP	Cerebral Palsy
CT	Computed Tomography
FAT	Femoral Antetorsion
FPA	Foot Progression Angle
IR	Internal Rotation
MRI	Magnetic Resonance Imaging
NS	Not Significant
PRISMA	Preferred Reporting Items for Systematic Reviews and Meta-Analyses
